# Preoperative Cortical Mapping for Brain Tumor Surgery Using Navigated Transcranial Stimulation: Analysis of Accuracy

**DOI:** 10.3390/brainsci14090867

**Published:** 2024-08-28

**Authors:** Wellingson Silva Paiva, Erich Talamoni Fonoff, Rhuann Pontes dos Santos Silva, Lucas Schiavao, André Russowsky Brunoni, César Cimonari de Almeida, Carlos Carlotti Júnior

**Affiliations:** 1 Neurosurgery Division, University of São Paulo, São Paulo 14040-906, Brazil; wellingsonpaiva@usp.br (W.S.P.); erich.fonoff@gmail.com (E.T.F.); schiva8@icloud.com (L.S.); brunoni@usp.br (A.R.B.); cesar.cimonari@gmail.com (C.C.d.A.); carlos.carlotti@hc.fm.usp.br (C.C.J.); 2 Medicine Department, Catholic University of Pernambuco, Recife 50050-900, Brazil

**Keywords:** brain neoplasms, motor cortex, transcranial magnetic stimulation

## Abstract

Transcranial magnetic stimulation (TMS) represents a distinctive technique for non-invasive brain stimulation. Recent advancements in image processing have enabled the enhancement of TMS by integrating magnetic resonance imaging (MRI) modalities with TMS via a neuronavigation system. The aim of this study is to assess the efficacy of navigated TMS for cortical mapping in comparison to surgical mapping using direct electrical stimulation (DES). This study involved 30 neurosurgical procedures for tumors located in or adjacent to the precentral gyrus. The DES points were compared with TMS responses based on the original distances of vectorial modules. There was a notable similarity in the points obtained from the two mapping methods. The distances between the geometric centers of TMS and DCS were 4.85 ± 1.89 mm. A strong correlation was identified between these vectorial points (r = 0.901, *p* < 0.001). The motor threshold in TMS was highest in the motor cortex adjacent to the tumor compared to the normal cortex (*p* < 0.001). Patients with deficits exhibited excellent accuracy in both methods. In view of this, TMS demonstrated reliable and precise application in brain mapping, which is a promising method for preoperative functional mapping in motor cortex tumor surgery.

## 1. Introduction

The oncological neurosurgery scope is to understand the anatomical and functional relationships between a brain tumor and the surrounding structures in order to propose a surgical strategy that allows for the maximum possible volume of lesion resection without causing damage to eloquent areas and, consequently, irreversible neurological deficits [1,2]. One of the major challenges in achieving this goal arises from the mass effect and tumor–brain interactions that trigger central nervous system (CNS) adaptation through neuroplasticity to maintain neurological and cognitive functions [3]. To address this, techniques for tumor tissue localization and identification have been developed and refined throughout history, with a focus on pre- and intraoperative mapping and monitoring of motor and language function [4].

Direct electrical stimulation (DES) remains the gold standard for minimizing postoperative complications during surgery but its method does not aid in formulating a pre-surgical plan [1,5]. In this regard, techniques such as navigated transcranial magnetic stimulation (nTMS) have been validated as effective pre-surgical functional mapping tactics for the motor cortex. This specific technique has a plausible advantage over functional magnetic resonance imaging (fMRI) and magnetoencephalography (MEG) as it does not require the patient to perform any voluntary tasks, but further studies are needed [6].

The nTMS technique utilizes a neuronavigation system to position a coil on the scalp, emitting magnetic pulses that induce an electric current to stimulate the cortical area and map the brain, enabling the investigator to visualize and distinguish between functional and non-functional cortical areas [1,5,7]. Motor mapping with nTMS is used for identifying the primary motor cortex and offers several advantages over DES, such as the possibility of repeated non-damaging tissue mapping, similar preoperative planning accuracy as well as being associated with longer progression-free survival (PFS) and better surgical outcomes [7,8,9,10].

Although widely used in recent decades, most published studies on the validity of nTMS use the distance between the “hotspot” and the nearest tumor margin. The aim of this study is, therefore, to report the application of preoperative nTMS and its spatial correlation with intraoperative cortical mapping based on DES during the resection of neoplastic CNS lesions located adjacent to or in the motor cortex, using the distance between the vector geometric center and the nearest tumor margin as a comparison.

## 2. Materials and Methods

### 2.1. Population

This prospective study included twenty-eight patients diagnosed with neoplastic lesions of the CNS adjacent to or in the motor cortex. All patients underwent magnetic resonance imaging at the Institute of Psychiatry of the University of São Paulo. For inclusion in this study, patients had to have the condition for outpatient follow-up, and if there were systemic lesions, they should be controlled during the evaluation period. Patients with Karnofsky Performance Scale <60 and aged over 80 years or under 18 years were excluded from the study, as well as those with multiple brain lesions and those with claustrophobia or unable to undergo magnetic resonance imaging or transcranial magnetic stimulation. All patients were treated by the same neurosurgeon (W.S.P.). Patients underwent a neurological evaluation before and after surgery, and regular follow-up was maintained for a minimum period of 3 months postoperatively. All patients signed an informed consent form agreeing to participate in the research, and the study was approved by the Ethics Committee (CAPPesq—Ethics Committee for Analysis of Research Projects at the Faculty of Medicine of the University of São Paulo, São Paulo, Brazil) and based on the recommendations established in the Helsinki Declaration.

### 2.2. Structural Imaging

High-field magnetic resonance imaging (1.5 T) was performed to correlate anatomical details with magnetic stimulation variables, intraoperative stimulation, and postoperative evolution. The protocol included the following sequences: (1) T1-weighted sequence with TR 1980 ms and TE 3.93 ms; and (2) T2 sequence. A 3D image of the brain was processed from the MRI to the navigation software. Both sequences used the following parameters: slice thickness of 1.0 mm, matrix size of 256 × 256, and a field of view (FOV) of 256 mm.

### 2.3. Transcranial Magnetic Stimulation Mapping

We used the Brainsight TMS Navigation system for mapping the motor cortical areas. This device was coupled to a flat surface figure-of-eight coil that delivered magnetic pulses through a MagPro X100 pulse generator (Magventure A/S, Farum - Denmark, 70 mm in diameter; peak magnetic field, 2.2 T; peak electric field, 660 V/m). The series of high-field magnetic resonance images as described above were transferred to the navigation system, which recreated multi-planar sagittal, coronal, and three-dimensional models. The planning of a minimum area to be stimulated was performed, consisting of a 36-point plane over the curvature of the cerebral convexity, covering the tumor and the precentral gyrus. nTMS was performed 1 to 2 days before surgery.

During the procedure, the patient was placed in a sitting position on a comfortable stretcher with head support. Surface electrodes were attached to the skin covering the thenar muscle (abductor pollicis brevis) for recording electromyography and monitoring the motor response to stimuli. Motor evoked potential (MEP) values were captured, and resting motor thresholds were determined. The previously defined minimum area of 36 points was then systematically stimulated, varying the pulse intensity until a response had at least 5 MEPs for 10 stimulations. The resting motor threshold was defined as the minimum stimulus intensity capable of producing at least 5 MEPs above 50 microvolts.

Motor mapping was then performed at 120% of the resting motor threshold on both the lesion side and the normal motor cortex. The points showing an evoked response were recorded (4 to 23 points were obtained in the study patients), using the intercommissural plane as a reference to define coordinates in the antero–posterior, lateral–lateral, and cranio–caudal axes (X, Y, and Z, respectively). A comparison between motor thresholds obtained in both cerebral hemispheres was made.

From the definition of the coordinates, the geometric center was calculated by vector summation of the three coordinates after weighting by the evoked potential. Vector magnitude calculation formulas and the Euclidean metric were used to calculate the magnitude of each mean vector in magnetic mapping. The Euclidean metric was also used to define the difference between these vectors and thus calculate the accuracy of the nTMS mapping method compared to direct cortical electrical mapping, defined as the gold standard.

### 2.4. Surgical Procedure

All patients were anesthetized intravenously with a bolus of propofol (1–2 mg/kg) and fentanyl (5–10 mg/kg). Analgesia was achieved with fentanyl(1–2 mg/kg). Anesthesia was maintained by continuous administration of propofol (75–125 mg/kg). Twenty minutes before the start of intraoperative cortical mapping, propofol was discontinued, and fentanyl was reduced.

Surgery was performed using a neuronavigation system for skin incision planning, followed by craniotomy marking. A straight line was made for the skin incision, marking the dimension capable of allowing a craniotomy with exposure of the motor cortex and tumor lesion. A drill was used for a single trephination and a craniotome (Midas rex Legend, Medtronic, MN, USA) for the craniotomy. After craniotomy and dura mater exposure, the precentral gyrus and the tumor were located with the aid of neuronavigation, and a navigation tool was attached to the bipolar stimulation electrode pen and registered. Stimulation parameters varied for each patient, with amplitudes between 1 and 10 mA; frequencies close to 60 Hz; pulse durations between 60 and 100 milliseconds; and a stimulus time of 1 s. Stimulation was performed by a surgeon who had no prior knowledge of the map obtained in nTMS, stimulating the entire exposed cortical surface; the locations where the desired motor response was provoked were acquired for map construction.

Resection of the tumor lesion was performed after mapping, using techniques and instruments traditionally used in microsurgical resection.

### 2.5. Statistical Analysis

Tumor characteristics were described using absolute and relative frequencies. Measures were described for each coordinate defined in the intercommissural plane, and the vector magnitude was calculated according to each measurement method. Intraclass correlations were calculated, with their respective 95% confidence intervals between methods, to assess random error. Bland–Altman plots were created to evaluate systematic error between each coordinate measurement and vector magnitude.

Absolute distances were created between each coordinate and the vector magnitude, and distances were described according to each tumor characteristic. Distances were compared between characteristics using Student’s *t*-test, except for histological type, where the absolute distances of coordinates and the vector magnitude between methods were compared using ANOVA.

Motor thresholds were described in patients and compared between sides using Student’s paired *t*-test. Pearson correlations were calculated between motor thresholds and absolute distances to verify the existence of a relationship between the measures.

Kolmogorov–Smirnov tests were applied to assess whether the probability distribution of the data is normal, with all data meeting this assumption (*p* > 0.05). Tests were performed at a significance level of 5%.

## 3. Results

Of the twenty-eight patients, two of them underwent two surgeries during the study period, totaling thirty surgeries in 24 months, with a minimum follow-up time of 3 months. Among the total participants, nine were female, and nineteen were male, with an average age of 45.66 +/− 14.62 years, ranging from 18 to 70 years (Table 1).

### 3.1. Clinical Findings

Regarding the pathological analysis, all patients presented one of the four anatomopathological tumor diagnoses. Nine surgeries were performed on patients with a diagnosis of glioblastoma multiforme, eight patients had low-grade gliomas, seven had meningiomas grade I, and six patients had cerebral metastases. Within the glioblastoma group, six were primary and two were secondary. Among the low-grade gliomas, five were Diffuse Astrocytomas, while three had characteristics of Oligoastrocytomas. In the meningioma group, all had grade I lesions. In the group of patients with metastases, four patients had primary lesions in the lung, one in the gastrointestinal tract, and one patient with melanoma. In eight (26.66%) patients, cortical mapping revealed functional areas invaded by the tumor, resulting in the termination of resection to prevent permanent deficits in the postoperative period. Regarding the extent of resection, complete macroscopic resection was performed in twenty-two surgeries, subtotal resection in six, and only biopsy in two cases. Complete resection was found in all meningioma cases, six patients with metastases, seven surgeries in patients with glioblastoma multiforme, and two patients with low-grade gliomas. Subtotal resection was found in two patients with glioblastoma multiforme and four patients with low-grade gliomas. In two patients with low-grade gliomas, only biopsy was possible due to significant motor activity throughout the tumor confirmed during cortical electrical stimulation. Epilepsy was present in 14 patients, and it was the initial symptom that led to the diagnosis of the disease in 11. Twenty-five patients were on anticonvulsant medication, with twenty-two using phenytoin, one using valproic acid, and two patients using oxcarbazepine.

### 3.2. Adverse Effects during the Surgical Time and Post-Surgery

In one patient, a paroxysmal event compatible with a simple partial seizure with a sensory component was observed during EMT [electromagnetic mapping], which spontaneously reverted. During DES [direct electrical stimulation], seizure activity was observed in 5 out of 30 patients, with no correlation with the intensity of the current used in the stimulation or a history of epilepsy. Surgical complications occurred in three (10%) patients in total, with wound dehiscence in one (3.33%), corrected with simple suture after returning to the emergency room; bacterial meningitis in one (3.33%); and pulmonary venous thrombosis in another patient (3.33%), treated with anticoagulation. During transcranial magnetic stimulation, headaches were observed in two patients treated with analgesics (500 mg oral dipyrone), and one patient experienced a simple partial seizure with a sensory component with spontaneous remission (Table 1).

### 3.3. Brain Mapping and Tumor Characteristics

Transcranial magnetic stimulation mapping was performed in all patients, obtaining 4 to 23 points (average 15.87 +/− 5.27 points) with an evoked motor response in the 30 mappings, totaling 476 points with a motor response out of a total of 2492 stimulated points. On average, 93 stimuli were applied, ranging from 64 to 119 per patient. Direct cortical stimulation mapping was also possible in all surgeries, although five patients (16.7%) experienced seizures during stimulation. In this intraoperative mapping, we obtained 4 to 14 points with motor response (average 8.87 +/− 2.16 points).

Regarding the accuracy of the mapping method with transcranial magnetic stimulation compared to electrical stimulation, we compared the difference in vector module values for the three coordinates according to the histological subgroup of patients. Analyzing these three coordinates, we did not identify any statistically significant differences between the different histological types, confirming the accuracy of mapping with EMT in the three spatial planes, regardless of the type of neoplasm (Table 2).

The vectorial differences by the Euclidean metric were assessed, and there was a smaller distance between vector modules found in patients with less malignant intrinsic tumors, specifically low-grade gliomas; however, this difference was not statistically significant (Table 2).

Analyzing the anatomical deformity generated by the tumor’s presence, it was found that in 18 patients, there was some degree of anatomical deformity in the precentral gyrus due to the tumor or edema (Table 2). Evaluating the effect of this displacement on the accuracy of the mapping method by EMT compared to mapping by electrical cortical stimulation (ECD), it was identified that the presence of this deformity, although it interfered with one of the coordinates, did not modify the vectorial distance in the two methods, maintaining the congruence of the vector modules (Table 2).

About the lesion location relative to the central sulcus, all studied patients had a maximum distance of 10 mm from the tumor to the central sulcus, characterizing a sample of lesions truly adjacent to this classic anatomical parameter of reference for the motor cortex. Stratifying into lesions with a distance less than 5 mm and lesions with a distance greater than 5 mm from the central sulcus, there was no interference in the coordinate module or vectorial distance in the different mapping methods (Table 2).

There was no statistical difference between the absolute differences of vectorial modules in X, Y, and Z coordinates when compared (Table 3): cortical (n = 21) and subcortical lesions (n = 9); intrinsic (n = 17) and extrinsic (n = 13); malignant (n = 23) or benign (n = 7) lesions; however, when it was compared, the distance of the tumor lesion to the motor cortex was less than 5 mm (n =12), and the second group had a distance greater than 5 mm (n =18).

Analyzing the clinical characteristics of the patients, 12 patients were operated on who presented some degree of motor deficit (Table 1). Of these, seven patients presented worsening of the deficit on the first day after surgery. Only two patients previously without deficits presented worsening in the immediate postoperative period, which was reversed after 3 weeks. In the late follow-up after 3 months, only one patient presented a worsening of the motor deficit compared to the preoperative period, characterizing it as a persistent deficit. The patient had a glioblastoma multiforme with a worsening of the deficit from grade IV to grade III. Of the twelve patients originally with deficits, eight showed improvement in the deficit, and three patients maintained the deficit at the three-month postoperative evaluation. In patients with previous deficits, the incidence of worsening in the immediate postoperative period was significantly higher than in those without deficits in the preoperative period.

The presence of preoperative deficit was analyzed with mapping accuracy, and it was found that although there is a difference in the vector module of the lateralization X coordinate, the final vector module distances were not significantly affected, maintaining the congruence of the two cortical maps (Table 3).

Regarding the previous surgical intervention during the study period, no change in the congruence of points was identified (Table 3). Although it modifies the X coordinate, there was no statistically significant modification in the vectorial distance of the main modules. Thus, the vector distance remained the same in patients operated on for the first time compared to reoperations, and the mapping with EMT remained accurate, even in patients with previous manipulation of the cerebral cortex.

Another clinical characteristic evaluated was the history of cerebral radiotherapy. Studying this characteristic, it was found that it modified the congruence in the Z coordinate but not the congruence of the vector distance, maintaining the accuracy of the EMT mapping method, regardless of this clinical characteristic.

Patients were subdivided into groups with resting motor thresholds with cortical electrical stimulation greater than 5 mA and lower than 5 mA. Seven patients presented lower thresholds, and twenty-three presented higher thresholds. There was no correlation between the degree of paresis and the motor threshold in ECD. In this variable, it was also verified that even in patients who required a higher current intensity for electrical mapping, the congruence of the vector modules of the mapping with EMT was maintained compared to mapping during surgery (Table 3). There was also a statistically significant difference in the motor thresholds between patients with meningiomas compared to other tumor groups.

**Figure 1:**
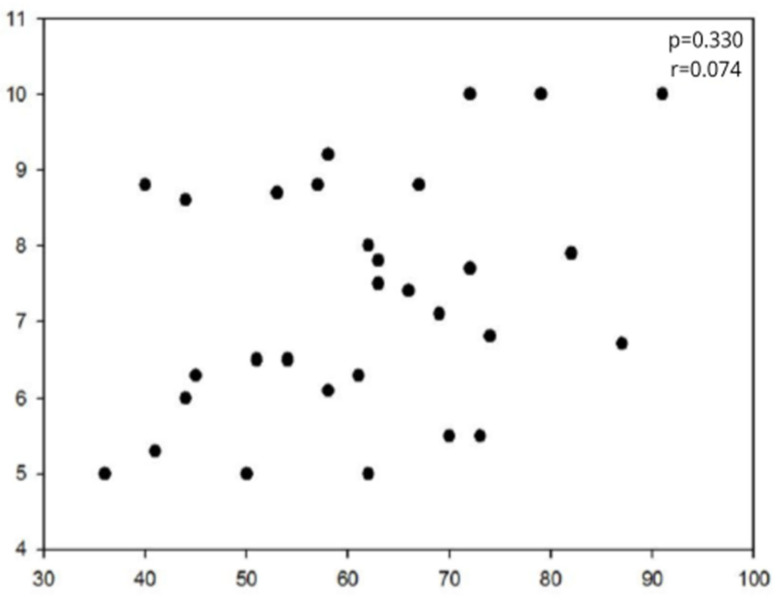
Correlation study between resting motor thresholds in nTMS, with the X-axis representing % of maximum generator activity, and the Y-axis representing threshold in DES (mA). nTMS: navigated transcranial magnetic stimulation; DES: direct electrical stimulation.

**Figure 2:**
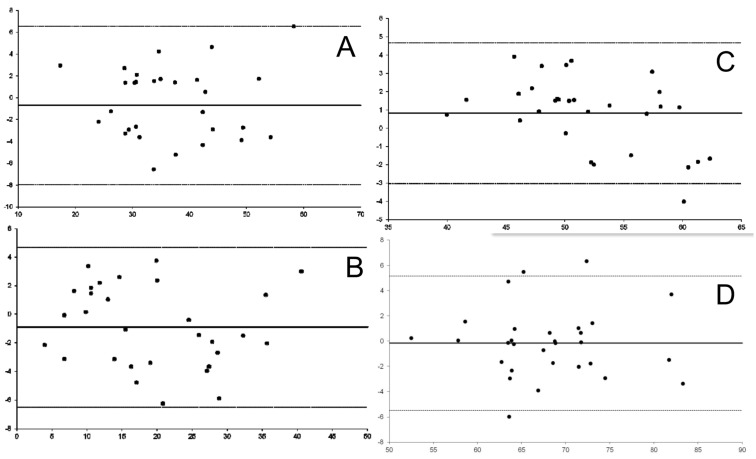
Bland–Altman distribution in the X (**A**), Y (**B**) Z (**C**) coordinates and vector magnitudes (**D**) between the two mapping methods. The X-axis displays the mean vector magnitude values for each patient. The Y-axis displays the vector difference for the vector magnitudes in nTMS comparing to DES. nTMS: navigated transcranial magnetic stimulation. DCS: Direct Cortical Stimulation.

The resting motor thresholds, when comparing the thresholds in magnetic stimulation with those obtained in cortical electrical stimulation, showed no correlation between the threshold values (Figure 1). The analysis of the distribution of vector module differences in the lateralization coordinate showed that in the Bland–Altman distribution, there was a trend toward 0 in the curve that measures the difference between the two methods, with an intraclass correlation tending to 1 (Figure 2A). Figure 2 does not show any trend in the differences between the methods for each coordinate or for the vector module, and the differences are randomly scattered around zero, indicating no systematic error between the methods. It was also observed that the increase in vectorial distance between the methods in the X coordinate is directly correlated with the deviations in the Z coordinate (r = 0.362, *p* = 0.050) and with the deviation in the vector module (r = 0.621, *p* < 0.001).

**Figure 3:**
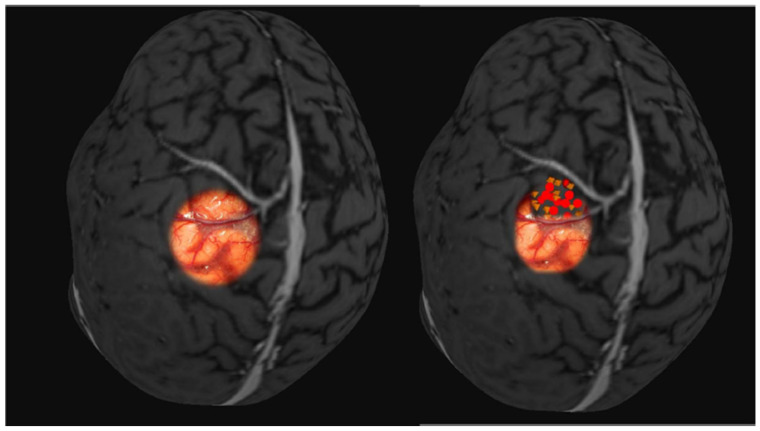
Three-dimensional MRI with points obtained in motor response in TMS (yellow octahedrons), and motor response recorded in the DES mapping (red circles) during the surgery. Fusion with intraoperative imaging. DES: direct electrical stimulation; MRI: magnetic resonance imaging; TMS: transcranial magnetic stimulation.

The average final Euclidean metric between the two mapping methods obtained a distance of 4.85 mm between the two vectors of the geometric center of the point cloud, which is the average distance between the two mapping methods. With a line tending to zero in the Bland–Altman distribution and correlation between the points with ICC = 0.901, *p* < 0.001, there was a strong correlation between the points obtained in the two mapping methods (Table 3, Figure 3). Evaluating the distance between the vector geometric center and the nearest tumor margin, a distance of 13.76 mm +/− 10.16 mm was found in magnetic stimulation compared to 13.28 mm +/− 10.31 mm in direct cortical electrical stimulation (r = 0.87, *p* = 0.01). The closest vector point was 0.71 mm in EMT, and the farthest was 28.34 mm. In direct cortical stimulation, the distances varied from 0.91 mm to 24.08 mm.

The resting motor thresholds in the two methods, when compared with different clinical characteristics, showed progressively higher motor thresholds in patients with greater peritumoral edema but without statistically significant difference (*p* = 0.0714). When comparing the degree of edema with the levels required for direct cortical stimulation to induce movement during intraoperative mapping, progressive levels of current intensity were observed, but the difference between the patients, although present (averages 6.3 and 8.4), was also not statistically significant (*p* = 0.183).

Evaluating the prognostic evolution of patients using the KPS scale pre- and postoperatively and the ECOG scale (Zubrod) pre- and postoperatively, a favorable evolution was observed in patients undergoing surgical treatment (Table 1). There was a proportion of improvement in the patients in the series according to KPS (*p* = 0.013). Using the Zubrod Scale to evaluate the proportion of improvement in patients in the preoperative and three-month postoperative period, a statistically significant improvement was also evidenced (*p* = 0.006). There was no correlation with tumor volume, epilepsy, and age; however, an association was found with the preoperative deficit and edema intensity.

## 4. Discussion

The most important finding shown was the similarity of the points performed in the two mapping methods. We found the distances between the geometric centers of TMS and DCS, with a strong correlation between these vectorial points. Furthermore, patients with deficits presented excellent accuracy in the two methods, and the clinical performance of the patients improved significantly 3 months after surgery. These findings provide direct evidence that preoperative nTMS motor mapping is comparable to intraoperative DES mapping in brain tumor patients. Even though DES mapping is the gold standard, nTMS provides valuable results about the brain cortex mapping.

Despite important advances in neuroimaging, functional information is necessary to better understand the spatial relationship between a tumor and the motor cortex [8,11]. nTMS is the only non-invasive preoperative mapping method that establishes a causative link between the stimulation of an area and the observation of a motor response similar to DES [9,12]. For this reason, the use of motor function in preoperative diagnostics seems more appropriate than functional imaging studies [13,14]. Moreover, navigated TMS (nTMS) allows mapping in patients who do not fit the criteria for functional magnetic resonance imaging (MRI) exams (young age, severe paresis, metallic implants, lack of cooperation, claustrophobia, or unconsciousness). The superiority of nTMS in preoperative evaluation compared to other imaging modalities has been described even in paralyzed patients [15]; however, this was a case report with one patient and included only nTMS and DES.

The first proposition to evaluate the accuracy of the method was initially proposed by correlating the nTMS map with the cortical map obtained with direct cortical electrical stimulation by Krings in 199716 and the cortical map obtained with nTMS compared to functional magnetic resonance imaging by the same study. In the United Kingdom, the first experience with the nTMS was reported by Jung et al., which showed that the nTMS resulted in a change in surgical strategy in 31.5% of patients with a brain tumor in eloquent areas [5]. Recent systematic reviews and meta-analyses show a reduced occurrence of postoperative permanent motor deficits, an increased GTR rate, and a tailored surgical approach compared to standard surgery without using preoperative nTMS mapping [8,9,16,17].

Other concurrent studies were conducted proposing an accuracy assessment of this mapping method. An observational study assessed 66 patients with motor-eloquent gliomas by nTMS combined with DES [18]. Mapping cartography of peritumoral motor areas was established for each patient. These authors reported in this prospective investigation that navigated nTMS allowed reliable and precise application of the magnetic pulse, and peritumoral somatotopy corresponded well between the two modalities in all 10 cases. The average distance between the tumor and the nearest point of the motor cortex was 7.9 mm (ranging from 5 to 15 mm; SD 3.2 mm) for nTMS and 6.6 mm (ranging from 0 to 12 mm; SD 3.4 mm) for DCES. Thus, these findings corroborate with our study, indicating accuracy of the methods for peritumoral somatotopy and therefore for surgical planning.

These findings are consistent with the most recent systematic review and meta-analysis, in which 8 of the 14 studies included assessed the motoric outcome comparing the two methods [19]. The nTMS was not inferior compared to DCS in terms of motoric outcome, which was marked subjectively by diamond location and objectively through a *p*-value of exactly 0.5. Another meta-analysis [9] with 14 studies included showed that nTMS influenced the surgical indications in 34.3–68.5%; in addition, another systematic review of 38 published studies showed that all studies reviewed concluded that nTMS correlated well with the “gold standard” of DES in patients with rolandic brain tumors [17].

Several methodological factors in the present study may have increased the similarity of results between nTMS and DES. First, there are always errors in the co-registration of the navigation system, but to compensate for this error, we repeated the navigation registration to avoid discrepancies and loss of accuracy, and during surgical navigation, an accuracy test was systematically performed every thirty minutes of navigation, and when there was a loss of accuracy, a new registration was repeated.

Another aspect to be highlighted is the potential loss of accuracy during intraoperative mapping as a result of brain displacement. To minimize this issue, we performed DES mapping at the beginning of surgery and made efforts to limit excessive cerebrospinal fluid leakage or brain swelling due to anesthetic complications, ensuring a rapid and careful dural opening. The accuracy found in our study can also be justified by the standardization of surgical mapping, as all patients were operated on by the same surgeon, ensuring consistency and adherence to a research protocol focusing on mapping, rather than mere surgical treatment without this emphasis.

The increased accuracy observed in our study is also attributed to the standardization of three accuracy calculation variables. We also did not use fixed preoperative points for mapping, thus excluding confounding variables within the anatomical margin. Stimuli were applied throughout the exposed region adjacent to the tumor. The results also indicate that the specified accuracy of this navigated transcranial magnetic stimulation (nTMS) system is achievable in a clinical setting. Unlike Forster and Picht, in our study, we performed freehand cortical mapping without constraints, stimulating the entire exposed cortex during the craniotomy [20,21].

Another variable compared to other studies is the stimulation intensity. We used 120% of the resting motor threshold (RMT). An intensity of 110% or 120% of the RMT would be sufficient to induce a motor potential in healthy volunteers. At 120% of the RMT, cortical inhibitory mechanisms are certainly triggered but short periods of muscle activation are avoided. All other mapping studies in tumors adjacent to the motor cortex were conducted with stimulation at 110% of the motor threshold [14,17]. This reveals that studies involving different coil orientations based on individual cortical anatomy are necessary, as well as various anatomical locations, especially when evaluating critical areas of cortical representation with low intensities.

Another unique aspect is the systematic evaluation of mapping methods, analyzing the accuracy of the mapping method according to different clinical variables, tumor characteristics, and cortical microenvironment, which presents its physiology modified by the presence of the tumor. To our knowledge, this is the first study in which navigated transcranial magnetic stimulation mapping is evaluated from the perspective of these different variables. We demonstrated that practically all conditions do not interfere with the accuracy of cortical maps. The peritumoral edema was the only studied variable that interfered in this accuracy. Studies report that mechanical and cellular changes can result in global changes in excitation and inhibition in the neuronal network, even outside of histologically significant cell lesions [22,23].

Although the accuracy of the mapping method was superior to other studies evaluating navigated transcranial magnetic stimulation and statistical similarity was found (with an intraclass correlation coefficient greater than 0.9), the approximately 4 mm that separates the vectors from the geometric centers of mapping still represent a significant distance for surgical resection definition. We attribute this difference to the loss in accuracy due to brain displacement after the opening of the dura mater, which, although efforts were made to avoid this, could still have interfered with the final result.

Another relevant aspect to highlight is that this vector difference concerns three-dimensional changes, including depth, and when put into a visual measure for the neurosurgeon, this vector difference becomes a smaller visual distance, making the method extremely accurate for clinical application in neurosurgery.

This accuracy is greater than that found in our study and even larger compared to previous studies. All these studies were conducted using “hotspots,” defined as the location of maximum excitability with the lowest stimulation intensity, as the mapping parameter. In our study, we did not use this parameter as we did not consider it ideal since it may undergo positional variation in pathological conditions [12,17,24,25,26].

Therefore, the geometric center of vector coordinates should offer greater accuracy regarding the real location of the motor cortex in transcranial magnetic stimulation, especially for pathological conditions like in our patients (with neoplasms, changes in cortical excitability, and structural deformity), as we consider it a more stable reference. Thus, we consider our investigation to be the first description of mapping comparisons using the most appropriate parameter for accuracy analysis.

Our results indicate that the specified accuracy of this navigated transcranial magnetic stimulation system is achievable in a clinical setting. The significant advantage of transcranial magnetic stimulation is that it is performed preoperatively, allowing for a more comprehensive and timely analysis of motor topography, including the evaluation of the real involvement of the tumor with the motor cortex, enabling surgical planning. Further studies should clarify the possibility of applying this method as a clinical mapping option to predict functional prognosis based on the degree of tumor involvement with the motor cortex.

## 5. Conclusions

This study demonstrates a congruence of points obtained in non-invasive mapping with navigated transcranial magnetic stimulation compared to points obtained in intraoperative mapping using direct cortical electrical stimulation. The nTMS technique spatially correlates well with the gold standard of DES, indicating significant accuracy between the two mapping methods. These findings can be helpful in planning the surgical strategy before the procedure to preserve eloquent areas in brain tumor patients.

## Figures and Tables

**Table 1:** Baseline characteristics of patients included. F = female; M = male; L = left; R = right; Y = yes; N = no; nTMS = navigated transcranial magnetic stimulation; KPS = Karnofsky performance status scale; ECOG PS = Eastern Cooperative Oncology Group performance status; PE = pulmonary embolism.

Patient	Sex	Age	Tumor Side	Initial Symptom	Preoperative MRC	Complication during nTMS	Intraoperative Seizure	Postoperative Complication	Preoperative KPS/ECOG PS
1	F	37	L	Seizure	V	N	N	N	100/0
2	M	39	R	Seizure	V	N	N	N	100/0
3	M	45	R	Headache	V	N	N	N	70/2
4	M	60	L	Motor deficit	I	N	Y	N	50/3
5	F	51	R	Headache	V	N	N	N	100/1
6	M	20	L	Seizure	V	N	N	N	100/0
7	F	57	R	Headache	III	N	Y	N	80/1
8	M	54	R	Headache	V	Headache	N	N	90/2
9	M	59	R	Motor deficit	III	N	Y	N	70/2
10	M	67	L	Mental confusion	III	Headache	N	PE	60/2
11	F	51	R	Motor deficit	IV	N	N	N	70/2
12	F	38	R	Headache	V	N	N	N	100/2
13	M	41	L	Seizure	V	N	Y	N	100/1
14	M	57	L	Motor deficit	III	N	N	N	60/3
15	F	24	L	Motor deficit	III	N	Y	N	80/2
16	M	43	R	Seizure	III	N	N	N	70/2
17	F	46	L	Headache	V	N	N	N	100/0
18	M	70	L	Motor deficit	IV	N	N	N	90/2
19	M	28	L	Motor deficit	V	N	N	N	100/0
20	F	59	R	Motor deficit	III	N	N	N	60/2
21	M	46	L	Seizure	III	N	N	Meningitis	80/2
22	F	24	L	Seizure	V	N	N	N	100/0
23	M	56	R	Motor deficit	III	N	N	N	80/1
24	M	59	L	Seizure	V	Seizure	N	N	100/0
25	M	34	R	Seizure	V	N	N	N	100/0
26	M	18	R	Seizure	V	N	N	N	100/0
27	F	34	R	Headache	V	N	N	N	100/0
28	F	32	L	Headache	V	N	N	Dehiscence	100/0
29	M	48	R	Seizure	V	N	N	N	100/0
30	M	70	L	Motor deficit	V	N	N	N	100/0

**Table 2:** Description of the absolute distances of the xX, yY, and Zz coordinates and the vector magnitude between the two methods according to clinical variables in the vicinity of the Pprecentral Ggyrus and the results of comparative tests. Bold values means the statistical significance (*p* < 0.05).

Variable	Difference in Magnitude at Coordinate Xx	Difference in Magnitude at Coordinate Yy	Difference in Magnitude at Coordinate Zz	Difference in Magnitude of the Vector
Tumor volume (< or >9 cm^3^)	0.333	0.61	0.401	0.116
Edema	**0.032**	0.606	0.103	**0.038**
Distance from the tumor to central sulcus < 5 mm (yes/no)	0.212	0.352	0.917	0.554
Involvement of Omega (yes/no)	0.51	0.417	0.888	0.36
Tumor involvement (cortical/subcortical)	0.558	0.079	0.975	0.544
Intrinsic tumors	0.9	0.966	0.072	0.745
Malignant/benign Ttumors	0.622	0.5	0.841	0.126
Distance from the tumor to the motor cortex < 5 mm (yes/no)	0.291	0.471	0.407	0.407
Preoperative deficit	**0.027**	0.654	0.219	0.057
Previous surgery	**0.031**	0.765	0.523	0.922
Previous radiotherapy	0.208	0.913	**0.036**	0.713
Threshold DES < 5 m (yes/no)	0.582	0.151	0.965	0.281

**Table 3:** Description of coordinates and vector magnitudes (Geometric Centers) in both measurement methods, average vector distances, standard deviation, and intraclass correlation results.

Coordinate	Mean	SD	N	ICC	CI (95%)		Absolute Mean Difference	SD Difference
					Lower	Higher		
Xm	36.63	9.72	30	0.893	0.753	0.938	4.2	2.08
Xe	37.34	9.79	30					
Ym	19	9.65	30	0.918	0.794	0.949	4.17	2.51
Ye	19.89	10,13	30					
Zm	52.52	5.42	30	0.897	0.731	0.94	3.42	2.01
Ze	51.69	6.37	30					
Vm Module	67.99	7.04	30	0.901	0.806	0.953	4.85	1.89
Ve module	68.14	7.09	30					

Xm: vector magnitude weighted by evoked potential of the laterality coordinate measured in mapping with transcranial magnetic stimulation; Xe: vector magnitude weighted by evoked potential of the laterality coordinate measured with direct cortical stimulation; Ym: vector magnitude weighted by evoked potential of the postero-anterior coordinate measured in mapping with transcranial magnetic stimulation; Ye: vector magnitude weighted by evoked potential of the postero-anterior coordinate measured with direct cortical stimulation; Zm: vector magnitude weighted by evoked potential of the depth coordinate measured in mapping with transcranial magnetic stimulation; Ze: vector magnitude weighted by evoked potential of the depth coordinate measured with direct cortical stimulation; Vm: vector magnitude weighted by evoked potential measured in mapping with nTMS; Ve: vector magnitude weighted by evoked potential measured in mapping with DES; SD: standard deviation; N: number; ICC: intraclass correlation coefficient; CI: confidence interval.

## Data Availability

The research data can be found in Library of University of São Paulo in https://www.teses.usp.br/teses/disponiveis/5/5138/tde-27072012-163513/publico/WellingsonSilvaPaiva.pdf (accessed on 5 March 2024).
